# Financial Preparation, Disaster Experience, and Disaster Risk Perception of Rural Households in Earthquake-Stricken Areas: Evidence From the Wenchuan and Lushan Earthquakes in China’s Sichuan Province

**DOI:** 10.3390/ijerph16183345

**Published:** 2019-09-11

**Authors:** Dingde Xu, Zhuolin Yong, Xin Deng, Yi Liu, Kai Huang, Wenfeng Zhou, Zhixing Ma

**Affiliations:** 1 Sichuan Center for Rural Development Research, College of Management of Sichuan Agricultural University, Chengdu 611130, China; 2 College of Management of Sichuan Agricultural University, Chengdu 611130, China; zhuolinyong@126.com (Z.Y.); 18583287370@163.com (Y.L.); 13247152095@163.com (K.H.); wenfeng970101@163.com (W.Z.); zhixingma66@163.com (Z.M.); 3 College of Economics of Sichuan Agricultural University, Chengdu 611130, China; xindeng66@126.com

**Keywords:** financial preparation, disaster experience, disaster risk perception, earthquake, Sihuan province, rural China

## Abstract

Sichuan is a province in Southwest China that is famous worldwide for its earthquakes. However, few quantitative studies in China have probed the correlations between rural households’ financial preparation, disaster experience, and disaster-risk perception. Using survey data of 327 rural households from four areas stricken by the Wenchuan Earthquake and Lushan Earthquake in Sichuan, the ordinary least square (OLS) method was used to quantitatively explore the correlations between these three factors. The results show that rural households’ total family cash income, asset diversity, and whether rural households can borrow money from relatives and friends whenever there is a catastrophe such as an earthquake are significantly negatively correlated with the probability of disaster occurrence. Asset diversity and whether rural households can borrow money from banks whenever there is a catastrophe such as an earthquake are significantly positively related to the severity of disaster occurrence. The severity of residents’ disaster experience is not significantly correlated with the probability of disaster occurrence, but is significantly positively related to the severity of the disaster. The research results can provide useful enlightenment for the improvement of financial preparedness and disaster risk management for rural households in earthquake-stricken areas.

## 1. Introduction

With the intensification of global climate change and geophysical movements since the beginning of the 21st century, earthquakes, tsunamis, landslides, mudslides, and other disasters have occurred frequently, profoundly influencing global economic and social development. Of these disasters, earthquakes are the most catastrophic, and thus, disaster-risk management for earthquakes has become a hot spot in academic and political circles [1,2]. According to Emergency Events Database (EM-DAT, the international disaster database), from 2000 to 2018, 87,877 people were killed and 8,303,867 people were affected by earthquakes worldwide [3].

China is a mountainous country with frequent earthquake disasters [4,5,6,7]. According to statistics, from 2000 to 2017, there were 179 earthquakes with a magnitude of 5 or above in China, resulting in 488,437 casualties and direct economic losses of 1,133,424.9 million Yuan [8]. As the world’s most famous earthquake province, Sichuan is the province with the most serious earthquake disasters in China. From 2000 to 2017, there were 18 earthquakes with a magnitude of 5 or above in Sichuan, leading to 459,993 casualties and direct economic losses of 938,710.93 million Yuan. Among them, the 5•12 Wenchuan Earthquake in 2008, the 4•20 Lushan Earthquake in 2013, and the 8•8 Jiuzhaigou Earthquake in 2017 were great earthquakes with a magnitude of 7 or above, causing 459,863 casualties and direct economic losses of 932,005.43 million Yuan [8]. The construction of a resilient disaster-prevention system in earthquake-prone residential areas has become a focus for government and academic circles. In general, however, academic studies of earthquake disaster-risk management are concentrated in developed countries (e.g., [9,10,11,12]), with insufficient attention given to the disaster-risk management of residents in earthquake-prone areas in China. In particular, studies of the disaster-risk management of rural households in mountainous settlements where earthquakes are extremely serious are relatively few. In China, due to the influence of geology and resource endowment, the settlements in mountain areas are not only disaster-prone areas, but also poverty concentrated areas. It is of great significance to pay attention to the disaster risk management of such groups, especially the risk response to the earthquake. Additionally, in the few researches on disaster risk management in earthquake-stricken areas in China, scholars pay attention to different contents due to different perspectives. For example, Cui et al. [13] mainly explores the construction of resilience and disaster prevention system at the community scale, Han et al. [14] mainly focuses on the impact of households with or without disabled members on their disaster preparedness. In general, few studies have focused on the correlation between financial preparedness and disaster risk perception from the perspective of rural household financial preparedness. Therefore, there is an urgent need to carry out such research [2,15].

Many empirical studies have shown that residents’ disaster preparedness is key to their coping with the impact of disasters [16,17,18,19]. However, the existing academic research mostly measures residents’ disaster preparedness from the perspective of ensuring the physical safety of residents. For example, residents are asked whether they have emergency supplies (such as flashlights, radios, and purified water), whether they have an escape plan, and whether they have purchased disaster-related insurance (e.g., [17,19,20]). However, in the face of earthquakes, which are highly catastrophic, such simple disaster preparedness as the preparation of an emergency kit can only guarantee the safety of residents to a certain extent and is of limited help in restoring and reconstructing communities after the devastation of an earthquake. In the face of earthquake disasters, national relief, social assistance, and catastrophe insurance can help residents carry out post-disaster reconstruction to some extent. However, from the three earthquakes with a magnitude of 7 or above in Sichuan in recent years, it can be seen that, compared with the direct losses caused by the disasters, external financial assistance was limited [15] and catastrophe insurance was not implemented. Thus, in the case of limited external relief funds, the post-disaster reconstruction of residents in earthquake-stricken areas had to rely on their own coping ability—namely, their financial preparation. Despite this fact, few academic quantitative studies have measured and explored the size and characteristics of residents’ financial preparation in earthquake-prone areas, thus, it is urgent to carry out this research [15]. Although the study on the characteristics of residents’ financial preparation in a small area has no obvious correlation with the residents’ financial preparation in the earthquake threatened area under other social and cultural backgrounds; however, the measure of financial preparation of rural households in earthquake-stricken areas in this study can provide useful enlightenment for disaster risk management in earthquake-threatened areas under other cultural backgrounds, and even make a comparative study of cross-regional and cross-cultural backgrounds. Meanwhile, China is a large earthquake country, and the earthquake sample region selected in this study is its typical representative region. Understanding the characteristics of rural households’ financial preparation in this region can provide certain reference basis for the implementation of financial preparation policies of rural households in other areas threatened by earthquake disasters in China. Facing the threat of earthquake disasters, rural households will take corresponding actions only when they perceive the risks [6,21]. Therefore, the disaster-risk perception of residents in disaster-prone areas and the influencing factors have always been the focus of academic research. The research by Lindell [18], Bubeck et al. [22], Huang et al. [23], Lindell and Perry [24], and Solberg et al. [25] has systematically reviewed residents’ disaster-risk perception and the driving factors as well as the correlations between disaster-risk perception and disaster-avoidance decisions. In the existing research, residents’ individual and family socio-economic characteristics (gender, age, years of education, residence time, occupation, etc.) and disaster experiences (whether they have experienced disasters before, whether they have incurred economic losses from disasters, etc.) are commonly used indicators. However, due to the differences in disaster types and regional socio-economic culture, the results of the correlations between these factors and residents’ disaster-risk perception are not uniform [22,24]. For instance, Lawrence et al. [26] have found that flood disaster experience can improve residents’ disaster-risk perception, and Xu et al. [21] have discovered that landslide disaster experience can enhance residents’ perception of the possibility of disaster occurrence but has no significant influence on the perception of threat and controllability of disaster occurrence. For earthquakes, which are especially serious as soon as they occur, how do residents’ socio-economic characteristics and disaster experience affect their disaster-risk perception? What are the correlations between financial preparation, which is an effective means for residents to cope with the impact of earthquake disasters, and disaster-risk perception?

In the above context, this study takes as its object rural households from areas stricken by the 5•12 Wenchuan Earthquake and the 4•20 Lushan Earthquake in Sichuan province (a major earthquake-prone province in China) and explores the construction of an index system for rural households’ financial preparation from the perspective of the internal- and external-coping abilities of these households. Moreover, it uses statistical models to explore the correlations between rural households’ financial preparation or earthquake disaster experience and disaster-risk perception. This study intends to solve the following two scientific problems:

(1) What are the characteristics of the financial preparation, disaster experience, and disaster-risk perception of rural households in earthquake-prone areas?
(2) What are the correlations between rural households’ financial preparation or disaster experience and disaster-risk perception in earthquake-prone areas?

## 2. Theoretical Development

The disaster-risk perception of residents in disaster-prone areas and the influencing factors have always been the focus of academic research. Scholars have conducted a lot of explorations on the correlations between residents’ individual and family socio-economic characteristics, disaster experience or disaster preparedness, and disaster-risk perception in different threat areas of different disasters, obtaining inconsistent results [27]. This study focuses on the correlations between residents’ financial preparation, disaster experience, and disaster-risk perception in earthquake-stricken areas in Sichuan.

### 2.1. Rural Households’ Financial Preparation and Disaster Risk Perception

According to existing studies, residents in disaster-stricken areas may experience the phenomenon of “survivor bias”, which means families with good financial preparation may underestimate the probability or impact of a disaster [15,22]. Therefore, families with financial preparation, such as residents with a high income diversity index, high income, high asset availability, deposits, and commercial insurance, generally believe that even if a major earthquake were to occur, they can reduce the impact of the disaster on their family by relying on the internal-coping ability of their family. For this reason, their perception of the severity of disaster occurrence is relatively low. For example, Lo and Cheung [15] have found that residents who purchase insurance have a relatively low perception of the severity of earthquakes. Miceli et al. [20] have discovered that residents’ disaster-preparedness behavior (including insurance purchase) is not significantly correlated with the probability of their risk perception of flood disaster occurrence, but it is significantly positively related to the severity of flood disaster occurrence. Xu et al. [19] have realized that residents’ insurance purchase behavior is significantly positively correlated with the threat and possibility of landslide disasters. Helweg-Larsen [28], Paton and Johnston [29] have found that individuals with disaster preparedness perceive a lower need for additional preparedness than other people.

Meanwhile, many empirical studies on disaster risk management show that the strength of residents’ social network also has an important impact on their risk perception and disaster preparedness [30,31,32]. Among them, in the face of the earthquake disaster, whether residents can smoothly borrow money from friends and relatives and borrow money from banks is an important sign of their social network. According to relevant researches in the field of sustainable livelihood (e.g., Reference [5]), rural households are faced with strong external shocks (including earthquake shocks), when their internal-coping capacity is insufficient to cope with the losses caused by the earthquake to their families, external-coping capacity (borrowing from relatives and friends and borrowing from banks) will come into play. Therefore, for families with different social networks, their disaster risk perception may present different states. For example, Lo and Cheung [15] have found that access to commercial credit is significantly positively correlated with the possibility of disaster occurrence. However, generally speaking, the maintenance of social network needs costs, thus, rural households will not easily use social network. Rural households only use them when they feel a strong threat and their internal-coping capacity is insufficient to cope with the threat. Therefore, the occurrence of severity perception and social network may be positively correlated. Based on this, research hypothesis H1 (including H1a,1b) was proposed.

**Hypothesis 1** **(H1).** *Rural households’ financial preparation is significantly correlated with disaster-risk perception. Specifically:*

**Hypothesis 1a** **(H1a).** *Rural households’ internal-coping ability is significantly positively related to the possibility of disaster occurrence, and is significantly negatively related to the severity of disaster occurrence.*

**Hypothesis 1b** **(H1b).** *Rural households’ external-coping ability is significantly negatively correlated with the possibility of disaster occurrence, and is significantly positively correlated with the severity of disaster occurrence.*

### 2.2. Residents’ Disaster Experience and Risk Perception

Residents’ disaster experience is often regarded as one of the important factors affecting risk perception. Although the measurement of residents’ disaster experience is not uniform [33,34], most empirical research results show that residents’ disaster experience is positively significantly correlated with their disaster risk perception. For example, Xu et al. [6,19] have used whether residents have landslide disaster experience to carry out the measurement, and have found that there are positive correlations between disaster experience and the possibility and severity of disasters. Lo and Cheung [15] have adopted the severity of residents’ disaster experience to carry out the measurement, and have found similar results. Based on this, research hypothesis H2 was proposed.

**Hypothesis 2** **(H2).** *Rural households’ disaster-experience severity is significantly correlated with disaster-risk perception. Specifically:*

**Hypothesis 2a** **(H2a).** *Rural households’ disaster-experience severity is significantly positively related to the possibility of disaster occurrence.*

**Hypothesis 2b** **(H2b).** *Rural households’ disaster-experience severity is significantly positively related to the severity of disaster occurrence.*

## 3. Data and Methods

### 3.1. Data Source

The data used in this study are mainly from the questionnaire survey conducted by the research team in the areas stricken by the Wenchuan Earthquake and the Lushan Earthquake in July 2019, and the survey method is a one-on-one face-to-face interview. The contents of the survey mainly include rural households’ sustainable livelihoods, residents’ disaster-risk perception, residents’ disaster-avoidance behavior, the construction of a resilient disaster-prevention system in villages, etc. The time for each questionnaire was about one and a half hours. Some objective indicators, such as rural households’ income, were mainly adopted to inquire about the family situation of rural households by the end of 2018, whereas some subjective indicators, such as residents’ disaster-risk perception, were primarily used to ask about the status of respondents when they were surveyed. In order to ensure the typicality and representativeness of the selected samples, this study chiefly adopted the stratified sampling method, which is a probability sampling technique, to determine the survey samples. The specific operation process was as follows:

Firstly, with regard to the selection of sample counties, this study was mainly based on the following two aspects:

1. The four sample counties should be from the areas stricken by the Wenchuan Earthquake and the Lushan Earthquake (each of the two major earthquakes involves two counties).
2. There are significant differences in economic development level between the two sample counties selected for each of the two major earthquakes.

Based on the above considerations, this study selected as sample counties Beichuan County and Pengzhou City from 10 counties stricken by the Wenchuan Earthquake (Pengzhou City is a county-level city), and Baoxing County and Lushan County from six areas stricken by the Lushan Earthquake.

Secondly, after sample counties were selected, two sample townships were randomly selected from each sample county according to differences in the level of economic development within the counties and the distance from the county center and the serious-disaster situation, especially the number of threatened people. A total of eight townships were obtained.

Thirdly, after the sample townships were determined, the villages in each sample township were divided into two types in accordance with the number of threatened people in the villages, the differences in the level of economic development, the distance from the township center, and other indicators; one village was randomly selected from each type of village as the sample village. In this way, a total of 16 villages were obtained.

Fourthly, concerning the determination of sample rural households, after the sample villages were determined, the front-line team members obtained the roster of rural households in the sample villages from village cadres, and 20–23 rural households were randomly selected from each sample village as sample rural households according to the pre-set random numerical tables.

Finally, in order to obtain high quality survey data, unify the questionnaire answer standard and teach the basic etiquette and skills of interview research, the teachers of the research group systematically trained 13 researchers who participated in the survey within one day; then, 13 researchers who received strict training conducted one-on-one face-to-face interviews in the homes of rural households under the guidance of village cadres, and a total of 327 valid questionnaires were obtained from 16 villages of eight townships in four counties.

### 3.2. Methods

#### 3.2.1. Selection and Definition of Model Variables

The objective of this study was to probe the correlations between rural households’ financial preparation, disaster experience, and disaster-risk perception. Rural households’ disaster-risk perception was the dependent variable of this study. With regard to the measurement of disaster-risk perception, in academic circles there are two main schools; namely, the cultural theory school (advocating qualitative study) and the psychological measurement paradigm (advocating quantitative study). Consistent with the research by Xu et al. [2], Armas [9], Xu et al. [21], Solberg et al. [25], Lindell and Whitney [35], Lo [36], Slovic [37], and Sun and Han [38], this study argued that disaster-risk perception is a measurable multi-dimensional concept. Based on this, this study mainly designed entries from the two dimensions of possibility and severity of earthquake disaster occurrence to measure disaster-risk perception, and the specific measurement entries are detailed in Table 1. Before the measurement, an internal consistency test was carried out on the entries, characterizing residents’ disaster-risk perception. Cronbach ɑ values corresponding to the probability of disaster occurrence, the severity of disaster occurrence, and the perception of disaster risks were 0.70, 0.64, and 0.72, respectively, which are all greater than 0.60, indicating that the entries designed by this study had good internal consistency. Subsequently, this study used factor analysis to reduce the dimensionality of the entry of disaster-risk perception, so as to obtain the two dimensions of the probability and severity of disaster occurrence. The value of Kaiser–Meyer–Olkin corresponding to factor analysis is 0.74, and the cumulative variance contribution rate of the two dimensions is 61.92%, which indicates that the results of the factor analysis are reasonable (Table 2).

Rural households’ financial preparation is one of the two core independent variables of this study. It relates to rural households’ income, family assets, or borrowing capacity. Based on this, referring to the setting of residents’ financial preparation in the research by Armas [9], Becker et al. [10], Lo and Cheung [15], Bubeck et al. [22], Keil et al. [39], Le Dang et al. [40], and Paul and Bhuiyan [41], this study mainly measured rural households’ financial preparation from two dimensions, which are the internal-coping ability and external-coping ability. Rural households’ internal-coping ability is characterized by total family cash income, income diversity, whether rural households have deposits, current asset diversity, and whether rural households purchase commercial insurance. Rural households’ external-coping ability is characterized by whether rural households can borrow sufficient money from formal institutions (such as banks) and whether they can successfully borrow money from informal channels (such as relatives and friends) when a catastrophe such as an earthquake occurs. Income diversity is measured by dividing the main income source channels of rural households by the total channels of income sources. The total channels of income sources mainly consist of five aspects: agricultural income, wage income, business income, income from relatives and friends, and transfer payment income (such as state subsidies), and the main income source channels of rural households are generally one or more of these five aspects. Current asset diversity is measured by dividing the present value of current assets by the present value of total family assets. The present value of current assets refers to the present value of valuable fixed assets owned by rural households, other than houses, while the present value of total family assets represent the present value of current assets as well as the present value of rural households’ housing assets.

Rural households’ disaster experience is the other core independent variable of this study. Mainly referring to the setting in the research by Lo and Cheung [15], this study adopts the severity of rural households’ earthquake disaster experience to measure rural households’ disaster experience.

In order to minimize the impact on the independent variables of missing important variables, this study, with reference to the research by Xu et al. [21], Bubeck et al. [22], Lindell and Perry [24], Lo [42], Lindell and Hwang [43], Sun and Sun [27], Yu et al. [44], Lindell and Perry [45], Lazo et al. [46], and Peng et al. [47,48], also includes some variables that may affect residents’ disaster-risk perception as control variables, mainly each respondent’s age, gender, years of education, occupation, residence time, nationality, etc.

#### 3.2.2. The Models

Since the probability of disaster occurrence and the severity of disaster occurrence, which are the dependent variables of this study, are interval variables, this study used ordinary least square (OLS) to estimate the models when exploring the correlations between rural households’ financial preparation or disaster experience and disaster-risk perception. The simple expression of the model is as follows:

$$Y_{i}=\alpha_{0}+\beta_{1i}\times FP_{i}+\beta_{2i}\times Experience_{i}+\beta_{3i}\times Control_{i}+\varepsilon_{i}$$

where ${\;Y}_{i}$ refers to the dependent variable of the model, which can be decomposed into two indicators, namely Possibility and Severity; $FP_{i}$, $Experience_{i}$, and $Control_{i}$ represent rural households’ financial preparation, disaster experience severity, and control variable, respectively; $\alpha_{0}$, $\beta_{1i}$, $\beta_{2i}\;$, and $\beta_{3i}$ represent the model parameters to be estimated, respectively; and $\varepsilon_{i}$ represents the residual term. Stata 11.0 was adopted in the whole model implementation process.

## 4. Results

### 4.1. Descriptive Statistics of the Variables

Table 3 shows descriptive statistics of the variables in the model. As for financial preparation, in terms of rural households’ internal-coping ability, rural households’ income diversity index was 0.5 on average, indicating that rural households have an average of 2–3 main income sources. The average annual family cash income is 66,238.94 Yuan, which showed great fluctuation. Among 327 sample rural households, 46% of them had deposits, and 28% of them had purchased commercial insurance. Lastly, rural households’ asset diversity index was 0.15 on average, indicating that rural households’ family assets were more allocated to real estate. With regard to rural households’ external-coping ability, whenever there was a catastrophe such as an earthquake, 69% and 75% of rural households could borrow money from banks and borrow money from friends and relatives, respectively. Concerning the severity of residents’ disaster experience, 89.60% of rural households believed that their disaster experience was serious. Regarding control variables, 46% of the respondents were women; the average age of the respondents was 53.44; the average years of education was 6.29; the average residence time at home was 41.71 years; 82% of the respondents were Han people; and 57% of rural households were farmers.

### 4.2. Model Results

Table 4 and Table 5 show the correlation coefficient matrix and regression analysis results of model variables, respectively. As shown in Table 4, the correlation coefficients among all variables of the models are below 0.8, which indicates that there is no serious multi-collinearity between the independent variables of the models. As shown in Table 5, Model 1, Model 2, and Model 3 are the results when the probability of disaster occurrence is taken as the dependent variable. Model 1 is the result of only including rural households’ financial preparation, and Model 2 is the result of only including the severity of disaster experience, while Model 3 is the result of including control variables based on Model 1 and Model 2. The results of Model 4, Model 5, and Model 6 are similar to those of Model 1, Model 2, and Model 3, except that the dependent variable of the models is the severity of disaster occurrence. From all the model test statistics (F value), it is seen that the results of all models, except Model 2, are significant at the level of 0.1 and above, and R-squared values corresponding to Model 3 and Model 6 are 0.113 and 0.107, respectively, suggesting that independent variables can, respectively, explain 11.3% and 10.7% of the probability of disaster occurrence.

As for the correlations between financial preparation and possibility in Model 1 and Model 3, rural households’ total family cash income, asset diversity, and whether rural households can borrow money from relatives and friends whenever there is a catastrophe such as an earthquake were significantly negatively correlated with the probability of disaster occurrence (Model 1). In other words, if rural households’ total family cash income is more, the proportion of the present value of current fixed assets in the present value of total family assets is larger, and rural households can borrow money from relatives and friends whenever there is a catastrophe such as an earthquake, rural households will believe that the probability of an earthquake will be smaller. Specifically, when other conditions remain unchanged, with every one unit increase in the logarithm of rural households’ total cash income, rural households’ perception of the probability of a disaster will decrease, on average, by 0.113 units. Furthermore, with every one unit increase in the proportion of the present value of current fixed assets in the present value of total family assets, rural households’ perception of the probability of disaster occurrence will decrease, on average, by 0.908 units. Lastly, compared with rural households who cannot borrow money from relatives and friends whenever there is a catastrophe such as an earthquake, rural households who can borrow money from relatives and friends in those circumstances will believe that the probability of a disaster will decrease, on average, by 0.292 units (Model 1). Meanwhile, it is noted that among the seven indicators of rural households’ financial preparation, four indicators (income diversity, whether rural households have deposits, whether rural households purchase commercial insurance, and whether rural households can borrow money from banks whenever there is a catastrophe such as an earthquake) were not significantly related to the possibility of disaster occurrence. The correlation coefficient between the severity of residents’ disaster experience and the possibility of a disaster is positive but not remarkable. In addition, all control variables (respondents’ gender, age, years of education, residence time, nationality, and occupation) were not significantly correlated with the probability of disaster occurrence.

As for the correlations between financial preparation and severity in Model 4 and Model 6, asset diversity and whether rural households can borrow money from banks whenever there is a catastrophe such as an earthquake were significantly positively related to the severity of the disaster. In other words, if the proportion of the present value of rural households’ current fixed assets in the present value of total family assets was larger, and rural households can borrow money from banks whenever there is a catastrophe such as an earthquake, rural households will believe that the severity of the earthquake will be stronger. To be specific, when other conditions remain unchanged, with every one unit increase in the proportion of the present value of rural households’ current fixed assets in the present value of total family assets, rural households’ perception of the severity of disaster occurrence will increase, on average, by 0.918 units. Compared with rural households who cannot borrow money from banks, whenever there is a catastrophe such as an earthquake, rural households who can borrow money from banks in those circumstances will believe that the severity of the disaster will increase, on average, by 0.245 units (Model 6). It is noted that among the seven indicators of rural households’ financial preparation, five indicators (i.e., apart from the above two indicators) are not significantly correlated with the severity of the disaster. If rural households’ earthquake disaster experience is more severe, their perception of the severity of the disaster will be stronger. Specifically, when other conditions remain unchanged, with every one unit increase in the severity of rural households’ earthquake disaster experience, rural households’ perception of the severity of the disaster will increase, on average, by 0.306 units (Model 6). Additionally, all control variables were not significantly related to the severity of the disaster.

## 5. Discussion

Rural households’ disaster preparedness is an important part of the construction of a regionally resilient disaster-prevention system, and financial preparation is the most important part of disaster preparedness, directly relating to the extent to which rural households can carry out post-disaster reconstruction. However, few studies have focused on the financial preparation of rural households in disaster preparedness, the measurement and characteristics of financial preparation of rural households in earthquake-stricken areas, and the correlation between financial preparation and disaster risk perception. The marginal contribution of this study was to make up for the above deficiencies. Specifically, this study constructed an index system for rural households’ financial preparation from the perspective of rural households’ internal-coping ability and external-coping ability; using survey data of rural households in the areas stricken by the Wenchuan Earthquake and the Lushan Earthquake, this study empirically analyzes the correlations between rural households’ financial preparation or disaster-experience severity and disaster-risk perception. The construction of a financial preparation index system and the results of the empirical research in this study can provide enlightenment for the construction of a resilient disaster-prevention system in earthquake-prone areas. Meanwhile, the measurement of financial preparation in this study can also provide useful enlightenment for other similar researches.

The internal-coping ability of rural households is an important factor affecting their disaster risk perception. Inconsistent with hypothesis H1a, this study found that income diversity, total cash income, whether rural households had deposits, and whether rural households purchased commercial insurance were not significantly correlated with residents’ disaster risk perception. At the same time, it is interesting that the correlation between asset diversity and the probability and severity of disasters turned out to be the opposite of research hypothesis H1a. The possible reasons for the results are as follows. The research area of this study is mostly hilly. Influenced by geographical location and natural resource endowment, rural households’ agricultural income or business income is not high. At the same time, with limited years of education and few skills, most migrant workers are engaged in construction and service industries, and their wages are relatively low. In the face of the impact of education, medical treatment of serious diseases, human relations, and other large expenditures, rural households’ annual cash income is not much. Therefore, deposits are relatively limited, and are generally insufficient to deal with the impact of earthquakes and other catastrophes on families. Meanwhile, there is no earthquake catastrophe insurance in the study area. While many rural households have purchased commercial insurance, this insurance is mostly used for old-age care or medical treatment for serious diseases, and thus, has little impact on rural households’ resistance to earthquakes. In addition, due to the expense of children’s marriages or competition with others in the village, who are also on a relatively low income, many rural residents in China generally invest the money they have saved over the years in fixed assets, especially housing (building fancy houses in their own villages or buying houses in county towns) [5]. Therefore, in this study, the asset diversity index is markedly correlated with residents’ disaster-risk perception. It is also noted that although many studies on sustainable livelihoods of rural households have shown that rural households can sell their fixed household assets to withstand external shocks (e.g., References [5,7]). However, earthquake shock is different from other risk shocks (such as medical expenses for serious diseases). If the earthquake shock is strong, it will directly threaten the lives and fixed assets of rural households. In this case, it will be difficult for rural households to sell their fixed assets to withstand the earthquake. At this time, rural households may have more survivor bias [15,22]. For the safety of life and assets, they will underestimate the possibility of disasters and overestimate the severity of disasters. Therefore, in this study, asset diversity is negatively significantly correlated with the probability of disaster occurrence, and positively significantly correlated with the severity of disaster occurrence.

In the face of external risk impact, when the internal-coping capacity of rural households is insufficient, their external-coping capacity will play a crucial role. Therefore, rural households’ external-coping capacity is also an important factor affecting their disaster risk perception and behavioral decision-making. Inconsistent with the hypothesis H1b, this study found that in the event of a major disaster such as an earthquake, the ability to borrow money from friends and family is negatively significantly correlated with the probability of disaster occurrence, but not significantly correlated with the severity of disaster occurrence. At the same time, in the event of major disasters such as earthquakes, the success of lending money to banks is positively significantly correlated with the severity of disaster occurrence, but not significantly correlated with the probability of disaster occurrence. The possible reasons for the results are as follows: the severity of the damage caused by earthquakes makes rural households more inclined to seek external help from relatives, friends, or banks when they are affected by disasters. When rural households are facing the serious impact of earthquake disasters, they cannot carry out recovery or reconstruction by only relying on their internal-coping ability, thus, they rely more on external support. However, the social networks of rural households are basically in the same county, and some are even concentrated in the same township or the same village. In the face of earthquake disasters, the relatives and friends of rural households may be affected as well. Thus, concerning the possibility of disaster occurrence, rural households are more inclined to seek help from relatives and friends when the internal-coping ability is insufficient, whereas with regards to the severity of disaster occurrence, rural households are more inclined to borrow money from banks when the internal-coping ability is insufficient.

Residents’ disaster-risk experience is another important factor affecting their disaster-risk perception. However, consistent with H2b, but inconsistent with H2a, this study finds that the severity of residents’ disaster experience is significantly positively correlated with the severity of disaster occurrence, but is not significantly related to the possibility of disaster occurrence. The results are different from the findings of Lo and Cheung [15]. Their research has found that residents’ disaster experience is significantly positively correlated with the possibility and severity of disaster occurrence. The possible reason is that the damage caused by these two major earthquakes is too serious, and residents in earthquake-stricken areas have a particularly deep memory of the severity of earthquake damage. Therefore, the severity of residents’ disaster experience is significantly positively related to the severity of disaster-risk perception. At the same time, earthquakes are low-frequency disasters that may occur only once in several decades. Although people are worried about the damage caused by earthquakes, they still believe that the possibility of recurrence in the next 10 years is relatively small (40.06% of the residents think that there will be no earthquake disasters in the next 10 years). Thus, in the research area, the severity of sample residents’ disaster experience is not significantly correlated with the possibility of disaster occurrence. Additionally, the differences between our study and Lo and Cheung [15] may also be due to the differences in sample areas and disaster risk perception measures. Lo and Cheung’s [15] disaster risk perception is measured by a single index, while this study uses factor analysis to reduce dimensions of multiple indexes. In the revised manuscript, we have also explained this situation.

In addition, interestingly, this study indicates that respondents’ gender, age, education level, nationality, job, and residence time are not significantly related to disaster-risk perception. The possible reason is that devastating earthquakes such as the Wenchuan Earthquake or the Lushan Earthquake have a great impact on people, and the people who have experienced the disaster have an extremely deep memory of the earthquake, thus, there are no remarkable differences in earthquake disaster-risk perception (the possibility and severity of disaster occurrence) among gender, age, education level, and other personal characteristics.

## 6. Conclusions and Implications

Based on the survey data of 327 rural households from four counties stricken by the Wenchuan Earthquake and the Lushan Earthquake in Sichuan province, this study analyzes the characteristics of rural households’ financial preparation, disaster-experience severity, and disaster-risk perception. Meanwhile, it constructs OLS models to probe the correlations between rural households’ financial preparation or disaster-experience severity and the perception of the possibility and severity of disaster occurrence. Three main conclusions are drawn:

(1) As for financial preparation, among 327 sample rural households, rural households’ income diversity index and asset diversity index are both less than 0.5, about one third rural households purchase commercial insurance, and less than half rural households have deposits; whenever there is a catastrophe such as an earthquake, about three quarters of rural households can borrow money from banks and can borrow money from friends and relatives.
(2) As for the correlations between financial preparation and disaster risk perception, the higher the rural households’ total family cash income, the higher the asset diversity, and when rural households can borrow money from relatives and friends whenever there is a catastrophe such as an earthquake, the lower their perception of the possibility of disaster occurrence, the higher the asset diversity. When rural households can borrow money from banks whenever there is a catastrophe such as an earthquake, the stronger their perception of the severity of disaster occurrence.
(3) As for the correlation between experience and possible disaster-risk perception, the more severe the disaster experience, the stronger their perception of the severity of disaster occurrence. Meanwhile, the severity of residents’ disaster experience is not significantly correlated with the probability of disaster occurrence.

Based on the above analysis, this study can also derive some useful policy implications. For instance, although this study found that many indicators of rural households’ internal-capacity are not significantly correlated with their disaster risk perception, they are also an important means for rural households to resist external risk impact (including earthquake disaster impact). This study found that the internal-capacity of rural households is not strong on the whole (e.g., only 28% of rural households have commercial insurance and 46% have deposits). The implication for the government is that the government should give more support to rural households, such as helping them find jobs and increasing family income through employment training, trying to implement earthquake catastrophe insurance to help rural households withstand the impact of earthquakes. In addition, this study indicates that rural households’ borrowing networks (i.e., borrowing money from relatives and friends and borrowing money from banks) are significantly correlated with their disaster-risk perception (the probability and severity of disaster occurrence), while the total cash income, income diversity, whether rural households have deposits, and whether rural households purchase commercial insurance and have financial preparation are not significantly related to disaster-risk perception. This reflects the importance of rural households’ social networks for post-disaster reconstruction. The message to the government is that it is necessary to pay attention to the construction of formal financial institutions and rural households’ borrowing networks and offer loan services for rural households in earthquake-stricken areas to help them carry out post-disaster reconstruction. At the same time, through reasonable guidance, there is a need to make full use of the support role of rural households’ social networks in their post-disaster recovery.

Despite its theoretical and practical significance, this study has some limitations, which can be improved upon in future research. For example, many empirical studies have shown that residents’ disaster-risk perception is significantly related to their disaster-avoidance behavior (e.g., [6,15,19,46,47,48]). However, this study only focused on the correlations between rural households’ financial preparation or disaster-experience severity and disaster-risk perception and did not explore rural households’ disaster-avoidance decisions. Future research, for example, can explore the correlations between rural households’ financial preparation or disaster experience and evacuation/relocation behavior decision/intention. For another example, in different empirical studies, the measurement criteria for disaster-risk experience are different, and this study uses the severity of residents’ disaster-risk experience, a relatively subjective indicator, to carry out the measurement. Hence, future research can consider using some objective and quantitative indicators to carry out the measurement and explore the influence of disaster-risk experience on residents’ disaster-risk perception or disaster-avoidance behavior.

## Figures and Tables

**Table 1:** Earthquake disaster risk perception measurement.

Entry Code	Dimension	Item ^a^	Mean	SD ^b^
A1	Possibility	In the next 10 years, there will be earthquakes near my home.	2.83	1.12
A2		I always feel that an earthquake will come one day.	3.08	1.32
A3		In recent years, the signs of earthquake disaster occurrence have become more and more obvious.	3.18	1.35
A4	Severity	I’m worried about the impact of an earthquake on the village and the Family.	4.19	1.12
A5		In the next 10 years, if an earthquake occurs, your and your families’ lives will be affected.	3.35	1.31
A6		If an earthquake occurs, the production and life of the villagers will be seriously affected.	4.16	1.06

Note: ^a^ 1 = totally disagree, 2 = disagree, 3 = neutral, 4 = agree, 5 = totally agree; ^b^ SD = Standard deviation.

**Table 2:** Component matrixes for respective components of risk perception after rotation.

Items	Components	
	Possibility	Severity
A1	0.84	0.09
A2	0.76	0.22
A3	0.71	0.05
A4	0.04	0.81
A5	0.10	0.80
A6	0.47	0.58
Eigenvalue	2.54	1.17
Explained variance	33.77%	28.15%
Cumulative variance	33.77%	61.92%
Cronbach ɑ	0.70	0.64

**Table 3:** Definition and descriptive statistics of the variables in the model.

Category	Variable	Definition and Measure	Mean	SD ^c^
Financial preparation	Income diversity	Income diversity = the main income source channels of rural households or the total channels of income sources	0.50	0.18
	Income	Total annual cash income of rural households (Yuan ^a^)	66,238.94	72,237.87
	Deposit	Whether rural households have savings (0 = no, 1 = yes)	0.46	0.50
	Insurance	Whether rural households buy commercial insurance (0 = No, 1 = Yes)	0.28	0.45
	Asset diversity	Asset diversity = the present value of current assets or the present value of total family assets	0.15	0.17
	Loans	Whether rural households can successfully borrow money from banks whenever there is a catastrophe such as an earthquake (0 = No, 1 = Yes)	0.69	0.46
	Borrow	Whether rural households can successfully borrow money from relatives and friends whenever there is a catastrophe such as an earthquake (0 = No, 1 = Yes)	0.75	0.43
Experience	Experience	The severity of residents’ disaster experience ^b^	4.56	0.76
Individual Characteristics	Gender	Responder gender (0 = male, 1 = female)	0.46	0.50
	Age	Responder age (year)	53.44	13.40
	Education	Years of education (year)	6.29	3.70
	Residence	Length of residence of responder (year)	41.71	19.78
	Nationality	Responder nationality (0 = other, 1 = Han)	0.82	0.39
	Occupation	Responder occupation (0 = other, 1 = Farmer)	0.57	0.50

^a^ 1 USD = 6.88 Yuan (at the time of the study); ^b^ 1 = very not serious, 2 = not serious, 3 = average, 4 = serious, 5 = very serious; ^c^ SD = Standard deviation.

**Table 4:** The correlation coefficient matrix of variables in the models.

Variables.	1	2	3	4	5	6	7	8	9	10	11	12	13	14	15	16
1. Possibility	1															
2. Severity	0.000	1														
3. Income diversity	−0.100 *	−0.063	1													
4. Ln(Income)	−0.166 ***	0.008	0.181 ***	1												
5. Deposit	−0.074	−0.036	0.065	0.213 ***	1											
6. Insurance	−0.107 *	−0.072	0.109 **	0.290 ***	0.140 **	1										
7. Asset diversity	−0.172 ***	0.121 **	0.129 **	0.067	0.090	0.063	1									
8. Loans	−0.016	0.094 *	0.010	0.223 ***	0.031	0.031	−0.080	1								
9. Borrow	−0.161 ***	−0.025	0.188 ***	0.194 ***	0.0650	0.107 *	0.017	0.117 **	1							
10. Gender	−0.043	0.069	0.035	0.038	−0.151 ***	0.082	0.016	−0.005	−0.059	1						
11. Age	0.130 **	−0.076	0.100 *	−0.226 ***	0.003	−0.132 **	−0.058	−0.209 ***	−0.066	−0.212 ***	1					
12. Education	−0.196 ***	−0.072	0.078	0.258 ***	0.172 ***	0.226 ***	0.051	0.171 ***	0.156 ***	−0.136 **	−0.496 ***	1				
13. Experience	0.059	0.227 ***	0.045	0.133 **	0.078	−0.029	−0.032	−0.001	−0.008	0.000	−0.017	−0.044	1			
14. Residence	0.166 ***	−0.035	−0.035	−0.129 **	−0.037	−0.159 ***	−0.025	−0.085	−0.143 ***	−0.268 ***	0.517 ***	−0.343 ***	−0.031	1		
15. Nationality	−0.088	−0.044	−0.071	0.0390	0.017	−0.010	−0.047	−0.038	−0.106 *	−0.06	−0.002	0.177 ***	−0.040	−0.036	1	
16. Occupation	0.101 *	0.033	−0.027	−0.263 ***	−0.102 *	−0.225 ***	0.062	−0.093 *	−0.084	0.102 *	0.271 ***	−0.371 ***	0.026	0.161 ***	−0.050	1

Note: *** *p* < 0.01, ** *p* < 0.05, * *p* < 0.1. 1–16, respectively, represent Possibility-Occupation; among them, 3–7 belong to internal-coping ability, while 8 and 9 belong to external-coping ability.

**Table 5:** Regression results of rural households’ financial preparation, disaster experience, and risk perception.

Variables	Possibility			Severity		
	Model 1	Model 2	Model 3	Model 4	Model 5	Model 6
1. Income diversity	−0.166		−0.197	−0.397		−0.379
	(0.309)		(0.314)	(0.314)		(0.315)
2. Ln(Income)	−0.113 *		−0.090	0.022		−0.019
	(0.060)		(0.063)	(0.061)		(0.063)
3. Deposit	−0.041		−0.039	−0.080		−0.066
	(0.111)		(0.113)	(0.113)		(0.113)
4. Insurance	−0.101		−0.018	−0.167		−0.125
	(0.126)		(0.129)	(0.128)		(0.129)
5. Asset diversity	−0.908 ***		−0.899 ***	0.869 ***		0.918 ***
	(0.326)		(0.327)	(0.331)		(0.327)
6. Loans	0.031		0.062	0.231 *		0.245 **
	(0.121)		(0.122)	(0.123)		(0.122)
7. Borrow	−0.292 **		−0.271 **	−0.047		−0.014
	(0.129)		(0.130)	(0.131)		(0.131)
8. Experience		0.078	0.082		0.299 ***	0.306 ***
		(0.073)	(0.072)		(0.071)	(0.072)
9. Gender			−0.088			0.073
			(0.121)			(0.121)
10. Age			−0.000			−0.006
			(0.005)			(0.006)
11. Education			−0.026			−0.027
			(0.019)			(0.019)
12. Residence			0.004			−0.001
			(0.003)			(0.003)
13. Nationality			−0.217			−0.025
			(0.144)			(0.144)
14. Occupation			0.053			−0.029
			(0.121)			(0.121)
Constant	1.666 ***	−0.359	1.192	−0.214	−1.365 ***	−0.737
	(0.607)	(0.339)	(0.785)	(0.618)	(0.330)	(0.786)
Observations	327	327	327	327	327	327
F	3.67 ***	1.13	2.84 ***	1.89 *	17.58 ***	2.68 ***
R-squared	0.074	0.003	0.113	0.040	0.051	0.107

Note: *** *p* < 0.01, ** *p* < 0.05, * *p* < 0.1. 1–14, respectively, represent an income diversity-occupation; among them, 1–5 belong to internal-coping ability, while 6 and 7 belong to external-coping ability.
